# Attention to effective treatment for Attention Deficit Hyperactivity Disorder (ADHD) - A data science project

**DOI:** 10.1192/j.eurpsy.2025.820

**Published:** 2025-08-26

**Authors:** Y. Bloch, L. Mosco

**Affiliations:** 1 child &Adolescent outpatient clinics & Research authority, Shalvata mental health center, Hod Hasharon; 2 School of Medicine, Tel Aviv university, Tel Aviv, Israel

## Abstract

**Introduction:**

ADHD (Attention-Deficit/Hyperactivity Disorder) is a common treatable disorder that impairs daily functioning along the life span. Pharmacotherapy plays a central role in managing ADHD, but adherence rates can be low, impacting treatment effectiveness.

**Objectives:**

To compare the adherence to the specific medication used to treat ADHD on specific patient populations.

**Methods:**

In this study, we used “Clalit Medical Services” anonymized data base and focused on the first year of treatment with the four available first-line pharmacotherapy products: Methylphenidate tablets, Methylphenidate Slow Release tablets, Methylphenidate Long Aacting capsules, and Oros Methylphenidate tablets. Analyzing data from 214,035 patients of all ages diagnosed with ADHD who initiated pharmacotherapy between 2000 and 2022, we used a Negative Binomial Regression to develop a model to predict the number of prescriptions purchased in the first year of treatment, serving as a proxy for adherence. Our main focus was on identifying medications that enable better adherence.

**Results:**

Oros Methylphenidatehad the highest number of predicted purchases (RR CI 95%: 5.85-5.96). After adjusting for calendar year effects, our results identified gender, age group, and socioeconomic status (SES) as significant predictors of adherence. A significant interaction effect revealed that the predicted number of purchases for a specific medication is influenced by the patient’s SES level, i.e., for the lower SES levels adherence with Methylphenidate was better than adherence with Oros Methylphenidate.

**Conclusions:**

The choice of the specific medication available as first-line treatment for ADHD, has a significant effect on adherence. Oros Methylphenidatehas has better adherence than the other MPH formulas. This would guide physicians to prefer the use of Oros Methylphenidateas first line therapy. This is not true for the lower SES. Strengthening our assumptions that knowledge about medication adherence and patient characteristics are potential indicators for improving the treatment of ADHD.

**Disclosure of Interest:**

None Declared

